# An Improved Stereo Matching Algorithm for Vehicle Speed Measurement System Based on Spatial and Temporal Image Fusion

**DOI:** 10.3390/e23070866

**Published:** 2021-07-07

**Authors:** Lei Yang, Qingyuan Li, Xiaowei Song, Wenjing Cai, Chunping Hou, Zixiang Xiong

**Affiliations:** 1 School of Electronic and Information, Zhongyuan University of Technology, Zhengzhou 450007, China; 2018006082@zut.edu.cn (Q.L.); 6745@zut.edu.cn (W.C.); 2 Dongjing Avenue Campus, Kaifeng University, Kaifeng 475004, China; 3 School of Electrical and Information Engineering, Tianjin University, Tianjin 300072, China; hcp@tju.edu.cn; 4 Department of Electrical and Computer Engineering, Texas A&M University, College Station, TX 77843, USA; zx@ece.tamu.edu

**Keywords:** image fusion, stereo matching, LNCC, STIF, vehicle speed measurement

## Abstract

This paper proposes an improved stereo matching algorithm for vehicle speed measurement system based on spatial and temporal image fusion (STIF). Firstly, the matching point pairs in the license plate area with obviously abnormal distance to the camera are roughly removed according to the characteristic of license plate specification. Secondly, more mismatching point pairs are finely removed according to local neighborhood consistency constraint (LNCC). Thirdly, the optimum speed measurement point pairs are selected for successive stereo frame pairs by STIF of binocular stereo video, so that the 3D points corresponding to the matching point pairs for speed measurement in the successive stereo frame pairs are in the same position on the real vehicle, which can significantly improve the vehicle speed measurement accuracy. LNCC and STIF can be used not only for license plate, but also for vehicle logo, light, mirror etc. Experimental results demonstrate that the vehicle speed measurement system with the proposed LNCC+STIF stereo matching algorithm can significantly outperform the state-of-the-art system in accuracy.

## 1. Introduction

Intelligent traffic surveillance is an important part of the intelligent transportation system. Intelligent traffic surveillance has provided vehicle speed measurement, traffic violation management, autonomous driving assistance, vehicle counting and classification [1,2,3,4]. Vehicle speed measurement plays an important role in intelligent traffic surveillance. Vehicle speed measurement methods can be divided into two groups: traditional speed measurement methods and video-based speed measurement methods [5,6]. Traditional speed measurement methods include induction loop speed measurement [7], ultrasonic sensor speed measurement [8], infrared sensor speed measurement [9], radar speed measurement [10]. For the induction loop method, the average speed is obtained by calculating the time interval the vehicle passes the two sensors with a fixed distance. The sensors need to be embedded beneath the road surface, and the installation and maintenance are complicated. For the other three methods, i.e., ultrasonic sensor, infrared sensor and radar, the speed are all calculated based on certain characteristics of the transmitted and received signals. However, these devices are easy to be detected due to the transmitted signals, which is undesirable for secret measurement. Video-based speed measurement has gained more and more attention because of its low cost, easy concealment and convenient combination of vehicle speed and vehicle information [11,12,13,14,15]. According to the video acquisition way, video-based methods can be further divided into two main categories: two-dimensional (2D) video-based method and three-dimensional (3D) video-based method.

The methods in [11,12] belong to 2D video-based speed measurement method. A vehicle speed measurement method based on pinhole imaging projection model combining frame difference with edge detection is proposed in [11]. The method in [12] is an improved version of the method in [11], which uses the shape-from-template technology to make the projection model more accurate and further improve the speed measurement accuracy. Nevertheless, the methods in [11,12] both utilize the principle of pinhole imaging, which is only suitable for speed measurement scenarios with vehicle traveling in a straight line. Moreover, the vehicle displacement calculated according to the plane projection relation is not accurate enough.

The methods in [13,14,15] belong to 3D video-based speed measurement method. A vehicle speed measurement method based on traditional object detection with image processing is proposed in [13], in which the vehicle target is detected by background subtraction. Speeded-up robust features (SURF) matching is performed on the vehicle target detected in the left and right view images, and the vehicle speed is estimated with the depth map. A vehicle plate speed measurement method based on WaldBoost classifier object detection is proposed in [14]. The vehicle plate is detected according to the local binary pattern (LBP) feature, stereo matching and 3D ranging are performed, hence the vehicle speed is calculated. A vehicle speed measurement method based on modern Convolutional Neural Network (CNN) object detection is proposed in [15]. An improved single shot multibox detector (SSD) network is used to detect the license plate, stereo matching and 3D ranging are performed, and the vehicle speed is calculated. This system cannot only secretly measure the speed of multiple vehicles traveling in multiple directions on multiple lanes, but also measure the speed of a vehicle in a curved or straight motion. Moreover, it can combine the vehicle speed measurement result with the vehicle characteristic. However, in the existing 3D video-based speed measurement methods, the optimization is mainly carried out on the object detection algorithm of the system, and the optimization is rarely carried out on the matching algorithm. The speed measurement accuracy of the system can be further improved.

The vehicle speed measurement method proposed in [15] is composed of three parts: vehicle characteristic detection, stereo matching and speed measurement. In the stereo matching process, a homography matrix is firstly used to remove the mismatching point pairs from the matching point pair set obtained by SURF. Then, a circular area is selected, respectively, as the constraint in the left-view and right-view images, with the center of the license plate as the center and the height of the license plate as the diameter. Only the matching point pairs that exist in both the left-view and the right-view circular areas are retained, and other matching point pairs are removed, by which the size of the matching point pair set is further reduced and the measurement efficiency is improved. Finally, the matching point pair closest to the license plate center is selected from the retained matching point pair set to represent the current vehicle position. In the process of calculating the homography matrix, four matching point pairs are randomly selected to perform the calculation. However, since the matching point pair set contains both correct matching and wrong mismatching point pairs, the error of the matrix would be very large if mismatching point pair exists in the four randomly selected matching point pairs, which will reduce the accuracy of speed measurement. Moreover, in the process of selecting the matching point pair closest to the center of the license plate as the measurement point, the matching point pairs selected in the consecutive frames may not correspond to the same position on the license plate, which will also reduce the accuracy of speed measurement due to the position difference of the measurement point.

In this paper, an improved stereo matching algorithm for the binocular stereovision-based vehicle speed measurement system in [15] is proposed. Firstly, the characteristic of license plate specification is transformed into a relationship between the pixel ratio of the license plate area in the image and the distance of the license plate to the camera. The matching point pairs with obviously abnormal distance to the camera are roughly removed from the matching point pair set obtained by SURF algorithm in the license plate area according to this relationship. Then, the mismatching point pairs are finely removed from the matching point pair set according to the LNCC, so as to further reduce the size of the matching point pair set. Finally, the best speed measurement point pair is selected by STIF of binocular stereo video. The matching point set obtained by SURF matching and LNCC mismatching removal on two consecutive left-view frames is taken as the temporal consistency constraint (TCC), so that the speed measurement point pairs in the consecutive frames correspond to the same position on the license plate. The two matching point sets, respectively, obtained by SURF matching and LNCC mismatching removal on the two consecutive stereo frame pairs are taken as the spatial consistency constraint (SCC), from which the two consecutive speed measurement point pairs are chosen. If the two points of a TCC matching point pair are, respectively, in the two consecutive SCC matching point sets, the corresponding SCC matching point pair is retained in a STIF matching point set. The STIF matching point pair closest to the center of the license plate is selected as the best speed measurement point. The proposed algorithm can significantly improve the accuracy of the license plate-based vehicle speed measurement system in [15]. In addition, the proposed stereo matching algorithm can be extended to other characteristics of the vehicle, such as logo, light and mirror, thus can also improve the accuracy of the optimized multi-characteristic-based vehicle speed measurement system.

The rest of the paper is organized as follows. In Section 2, we review some related works on matching. In Section 3, we propose an LNCC+STIF stereo matching optimization algorithm. In Section 4, we report the experimental setup and results. In Section 5, we make a conclusion.

## 2. Related Works

Image matching aims to identify the same or similar structure from two or more images. Image matching is widely used in computer vision [16], pattern recognition [17], medical image analysis [18], etc. It is the basis of image fusion [19,20]. Image matching methods can be divided into two categories: region-based methods and feature-based methods [21,22]. For the region-based methods, such as correlation-like method [23], Fourier method [24], and mutual information method [25], the image saliency information is provided by pixel intensity [26], which is neither suitable for image with few salient details, nor insusceptible to image distortion and illumination change. For the feature-based methods, salient features, such as points, lines and surfaces, are firstly extracted from the images, which are then used to achieve image matching. The extracted features cannot only represent the image structure better, but also reduce the impact of image quality reduction [27].

In the feature-based matching method, image matching can be classified into direct matching and indirect matching [28]. For direct matching, the correspondence between two given feature sets is established by direct utilization of spatial geometric relationship [29,30]. For indirect matching, the matching task is decomposed into two stages: (1) A matching point set is constructed by calculating the similarity between descriptors. Lowe [31] proposes a scale-invariant feature transform (SIFT) descriptor based on distance ratio, but with slow speed and heavy calculation burden. SURF [32] is an accelerated version of SIFT. However, mismatching will inevitably occur when constructing the matching point set by local features [33,34]. (2). Mismatching points are removed from the matching point set by additional constraints. Mismatching removal methods can be divided into three categories: resampling-based, non-parametric model-based and learning-based.

Resampling-based methods are widely used for automatic matching of remote sensing images [35]. Random sample consensus (RANSAC) is a classic resampling-based method, with several variants such as maximum likelihood estimation sample consensus [36] and progressive sample consensus [37]. These methods use a hypothesis-verification strategy. A hypothesis subset is selected to estimate the parametric model and the smallest non-outlier subset is obtained by repeated resampling. The resampling-based method relies on the preselected parametric model. The efficiency of the model is reduced when the image transformation is non-rigid. When the proportion of outliers in the matching set becomes large, the performance of these methods will degrade seriously [38].

Non-parametric model-based methods introduce more prior knowledge, such as motion consistency, and can handle degraded scenes. Different deformation function can be used to establish different models for different transformation. In [39,40], an estimator is used to model the deformation function. In [41,42], a guided locality preservation matching method is proposed to process the matching set with a large proportion of outliers, which only preserves the neighborhood structure of the potential correct matching between two images. Ma et al. converted the mismatching removal problem into a spatial clustering problem with outliers [43]. The initial matching set is divided into several clusters with motion consistency and one cluster with outliers. The matching performance in the case of serious data degradation is improved by iterative clustering strategy.

Learning-based methods are often used to extract and represent features. Learning-based matching can be divided into image-based learning and point-based learning. Image-based learning can be directly applied without detecting any salient image structures in advance [44]. Point-based learning is inclined to perform matching on the extracted point set [45]. Ma et al. converted the mismatching removal problem into a two-class classification problem. The classifier is trained based on a general match representation associated with each putative match through exploiting the consensus of local neighborhood structures based on a multiple K-nearest neighbors strategy [46].

## 3. Proposed Method

An improved stereo matching algorithm for the binocular stereovision-based vehicle speed measurement system in [15] is proposed in this paper. The proposed algorithm consists of two stages: mismatching removal optimization for vehicle characteristics, and best vehicle speed measurement point selection optimization.

**Figure 1:**
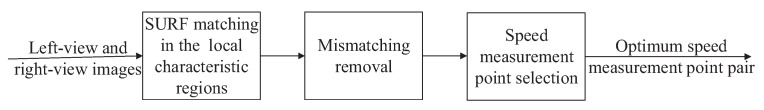
Matching algorithm flowchart.

The process of stereo matching in [15] can be divided into three steps: SURF matching in the detected local characteristic regions, mismatching removal, and speed measurement point selection. The flowchart is shown in Figure 1.

In the SURF matching process, only feature points in the license plate regions of the left-view and right-view images are matched in [15]. Not only the number of matching calculations is reduced, but also the interference from the feature points outside the license plate regions is avoided. Thus, the SURF matching in the local characteristic regions in [15] is reused in this paper.

In the mismatching removal process, the speed measurement system in [15] uses a homography matrix to eliminate mismatching point pairs from the matching point pair set obtained by the SURF matching. The homography matrix is calculated by randomly selecting four matching point pairs. However, the matching point pair set contains both correct matching and wrong mismatching point pairs. If mismatching point pair exists in the four selected matching point pairs, the error of the calculated matrix will be large, which will affect the accuracy of speed measurement. In this paper, the relationship between the pixel ratio of the license plate region in the image and the distance of the license plate to camera is fitted according to the characteristic of license plate specification. With this relationship, the matching point pairs with obviously abnormal distance to the camera are roughly removed from the matching point pair set obtained by SURF matching in the license plate regions. LNCC aims to preserve the potential local neighborhood structure of the correct matching. Therefore, more mismatching point pairs are finely removed from the matching point pair set in the license plate regions by LNCC. LNCC can also be used to remove mismatching point pairs from the matching point pair sets in the logo, light, and mirror regions, respectively.

In the speed measurement point selection process, the matching point pair closest to the center of the license plate is selected to represent the current vehicle position [15]. Nevertheless, there is no guarantee that the matching point pairs selected in the consecutive frames are at the same spatial location on the license plate. The spatial location difference between the speed measurement points will also reduce the speed measurement accuracy. In this paper, the best speed measurement points in the stereo video are selected by STIF. SURF matching is performed on two consecutive left-view frames and LNCC is used to remove the mismatching point pairs. The matching point pair set obtained on two consecutive left-view frames is taken as TCC, so that the speed measurement points selected from the consecutive frames are at the same spatial location on the license plate. SURF matching is performed on the left-view and right-view stereo images and LNCC is used to remove the mismatching point pairs. The matching point pair set obtained on the stereo images is taken as SCC. If the two points of a TCC matching point pair are, respectively, in the two consecutive SCC matching point sets, the corresponding SCC matching point pair is retained in a STIF matching point set. The STIF matching point pair closest to the center of the license plate is selected as the optimum speed measurement point.

### 3.1. Mismatching Removal Based on License Plate Specification Constraint (LPSC)

The license plate specification is settled by the vehicle management department, including the strict regulations on the size, color and content of license plates [47]. For the car used in the experiments of this paper, the size of the license plate is fixed, i.e., 440 mm × 140 mm. The closer the vehicle is to the camera, the larger the pixel ratio of the license plate region in the image.

A matching point pair set $S={\left\{\left(p_{li},p_{ri}\right)\right\}}_{i=1}^{N}$ is obtained by SURF matching on stereo image pair, wherein, $p_{li}$ represents the left-view matching point and $p_{ri}$ represents the right-view matching point. Mismatching point pairs exist in the set *S* and need to be removed. Since the license plate size is fixed, the relationship between the pixel ratio of the license plate region in the image and the distance of the license plate to camera is fitted. The matching point pairs with obviously abnormal distance to the camera are roughly removed from the set *S* according to this relationship.

The speed measurement range, that is, the distance between the vehicle and the camera is set to 1–15 m. The pixel ratio of the license plate in the image is calculated every 0.5 m, as shown in Table 1. When the distance is 15 m, the smallest ratio is 0.0416%. When the distance is 1m, the largest ratio is 8.1130%.

**Figure 2:**
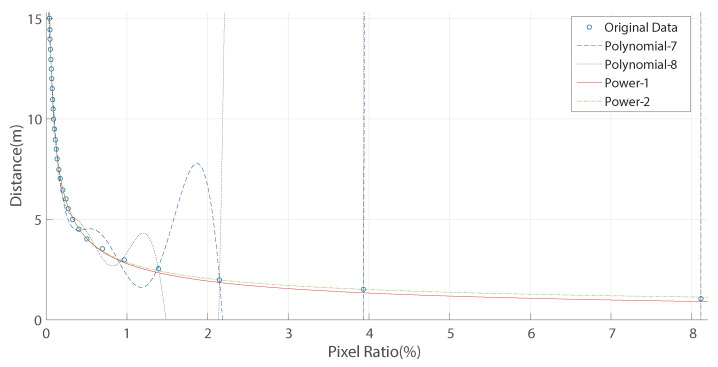
Fitting curves of four fitting functions.

To find the relationship between the pixel ratio of the license plate region in the image and the distance of the license plate to camera, two types of fitting function can be used: polynomial and power. The fitting effect can be evaluated with four parameters: RMSE, SSE, R-square, and Adj R-sq. RMSE represents the difference between the predicted value and the true value. The smaller the RMSE, the better the fitting effect [48]. The performance comparison of four fitting functions is shown in Table 2: Polynomial-7, Polynomial-8, Power-1, and Power-2. The fitting curves of the four fitting functions are shown in Figure 2.

In Figure 2, the hollow circle represents the actual measured data. The blue dotted line represents the fitting curve by Polynomial-7. The black dotted line represents the fitting curve by Polynomial-8. The red solid line represents the fitting curve by Power-1. The green dot-dash line represents the fitting curve by Power-2. The fitting curves by Polynomial-7 and Polynomial-8 is over-fitting, and thus are discarded. The fitting curves by Power-1 and Power-2 are similar, of good fitting effect. The R-square and Adj R-sq parameters of Power-1 and Power-2 are the same, while the SSE and RMSE parameters of Power-2 are smaller than that of Power-1. Therefore, the Power-2 function with better fitting performance is chosen to fit the relationship between the pixel ratio of the license plate region in the image and the distance of the license plate to camera, as shown in Equation (1):

(1) $$d=2.505*r^{-0.5651}+0.3637$$

wherein, *r* represents the pixel ratio of the license plate region in the image, and *d* represents the distance between the license plate and the camera.

When the measurement range is no more than 15 m, the ranging error is no more than 3% [49], which can be used as a mismatching removal condition. If Equation (2) is not met, the matching point pair is removed:

(2) $$\left|\frac{d_{match}-d}{d}\right|\leq 3\%\;$$

wherein, $d_{match}$ represents the distance from the matching point to the camera calculated by Zhengyou Zhang’s camera calibration method [50], and *d* represents the distance from the license plate to the camera calculated by the fitting function in Equation (1).

**Figure 3:**
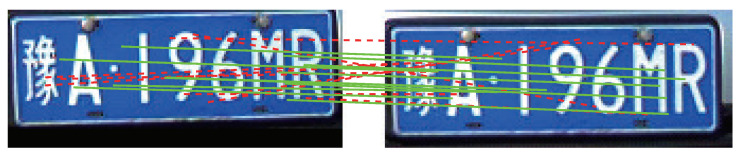
An exemplary matching result with LPSC-based mismatching removal.

Table 3 shows the comparison of matching point pair number with and without LPSC-based mismatching removal. With LPSC, the number of matching point pairs is significantly reduced. However, mismatching point pairs still exist in the reserved matching point pair set with LPSC, as shown in Figure 3. The green solid line represents the correct matching point pair. The red dashed line represents the wrong mismatching point pair. Several mismatching point pairs still exist and need to be further removed.

### 3.2. Mismatching Removal Based on LNCC

For license plate, mismatching point pairs still exist after SURF with LPSC. For logo, light and mirror, mismatching point pairs also exist after SURF. LNCC is used to further remove more mismatching point pairs, which aims to preserve the potential local neighborhood structure of the correct matching point pairs.

**Figure 4:**
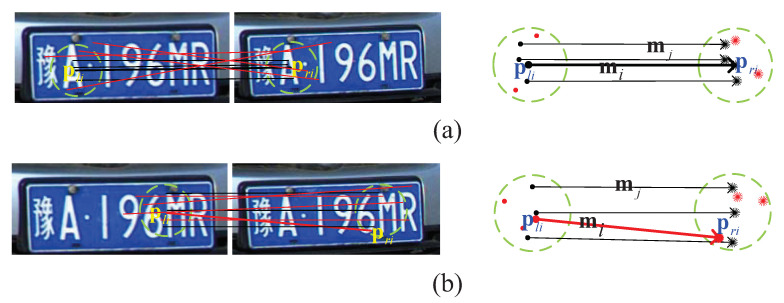
Exemplary neighborhood consistency diagrams. (**a**) An exemplary neighborhood consistency diagram of a correct matching point pair. (**b**) An exemplary neighborhood inconsistency diagram of a wrong mismatching point pair.

For the matching point pair $\left(p_{li},p_{ri}\right)$, other *n* pairs of matching point (n=3) located in both the neighborhood $N_{p_{li}}$ of $p_{li}$ and the neighborhood $N_{p_{ri}}$ of $p_{ri}$ are selected. Neighborhood $N_{p_{li}}$ and $N_{p_{ri}}$ are, respectively, composed of 5 neighbors with the nearest Euclidean distance in the corresponding point sets of $p_{li}$ and $p_{ri}$. As shown in Figure 4, the matching point pair $\left(p_{li},p_{ri}\right)$ is converted into a displacement vector $m_{i}$, with the starting point and ending point of $m_{i}$ corresponding to the right-view and left-view matching point $p_{li}$ and $p_{ri}$, i.e., $m_{i}=p_{ri}-p_{li}$. The difference between $m_{i}$ and other $m_{j}$ in its neighborhood is calculated to judge the neighborhood consistency, i≠j. Figure 4a shows an exemplary neighborhood consistency diagram of a correct matching point pair $\left(p_{li},p_{ri}\right)$, wherein $m_{i}$ and $m_{j}$ are in the same direction and of the same length. Figure 4b shows an exemplary neighborhood inconsistency diagram of a wrong matching point pair $\left(p_{li},p_{ri}\right)$, wherein $m_{i}$ and $m_{j}$ are in different directions and of different lengths.

The neighborhood consistency index between $m_{i}$ and $m_{j}$ is defined by Equation (3):

(3) $$C\left(m_{i},m_{j}\right)=\frac{\min\left\{\left|m_{i}\right|,\left|m_{j}\right|\right\}}{\max\left\{\left|m_{i}\right|,\left|m_{j}\right|\right\}}\cdot \frac{\left(m_{i},m_{j}\right)}{\left|m_{i}\right|\cdot \left|m_{j}\right|}$$

wherein (·,·) represents the inner product operation of two vectors, |·| represents the modulus operation of a vector, max{·,·} represents the maximization operation, and min{·,·} represents the minimization operation. $C\left(m_{i},m_{j}\right)\in \left[-1,1\right]$, and $C\left(m_{i},m_{j}\right)=1$ correspond to the highest the neighborhood consistency.

The number of matching point pairs whose $C\left(m_{i},m_{j}\right)$ is close to 1 is defined as $n_{C}$, $n_{C}\leq n$. If $n_{C}=3$, $m_{i}$ is consistent with three $m_{j}$ in its neighborhood, then $m_{i}$ is judged to be a correct matching point and retained. If $n_{C}=2$, $m_{i}$ is consistent with two $m_{j}$ in its neighborhood and inconsistent with the other one $m_{j}$ in its neighborhood, then $m_{i}$ is also judged to be a correct matching point and retained. If $n_{C}=1$, $m_{i}$ is consistent with one $m_{j}$ in its neighborhood and inconsistent with the other two $m_{j}$ in its neighborhood, then $m_{i}$ is temporarily retained and judged again in the second iteration. If $n_{C}=0$, $m_{i}$ is inconsistent with three $m_{j}$ in its neighborhood, then $m_{i}$ is judged to be a wrong mismatching point and removed.

**Figure 5:**
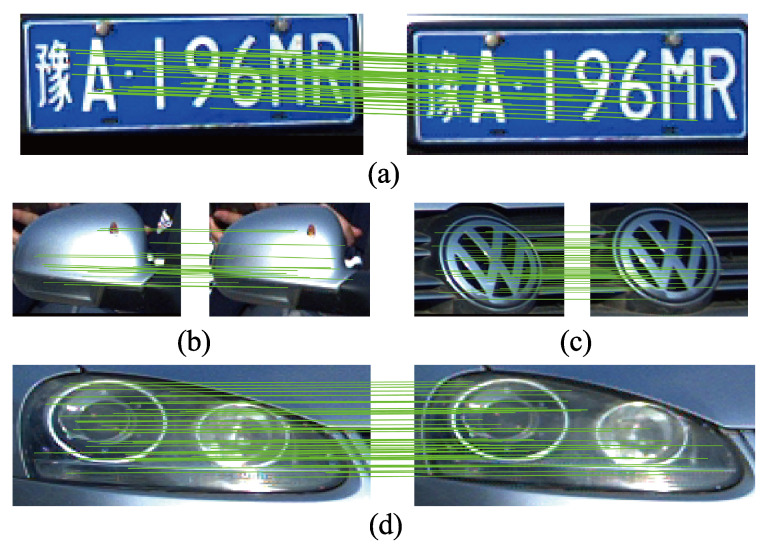
An exemplary matching result with LNCC-based mismatching removal. (**a**) License plate. (**b**) Mirror. (**c**) Logo. (**d**) Light.

Table 4 shows the comparison of matching point pair number with and without LNCC-based mismatching removal for license plate, logo, light and mirror, respectively. With LNCC, the number of matching point pairs for each vehicle characteristic is significantly reduced. Exemplary matching results with LNCC-based mismatching removal for license plate, logo, light and mirror are, respectively, shown in Figure 5. The solid green line represents the correct matching point pair.

### 3.3. Speed Measurement Point Selection Based on STIF of Binocular Stereo Video

For the vehicle speed measurement, not all the correct matching point pairs are needed, and only one optimum matching point pair needs to be selected from the matching point pair set obtained by SURF with LPSC and LNCC. In [15], the matching point pair closest to the license plate center is selected to represent the vehicle position in the current frame. However, this selection method cannot guarantee that the matching point pairs selected in two consecutive frames are at the same spatial position on the license plate. The spatial position difference between the speed measurement points will also cause speed measurement accuracy reduction. In this paper, a STIF-based speed measurement point selection method is proposed, which constructs a smaller matching point pair set with SCC and TCC, from which the speed measurement point is selected.

**Figure 6:**
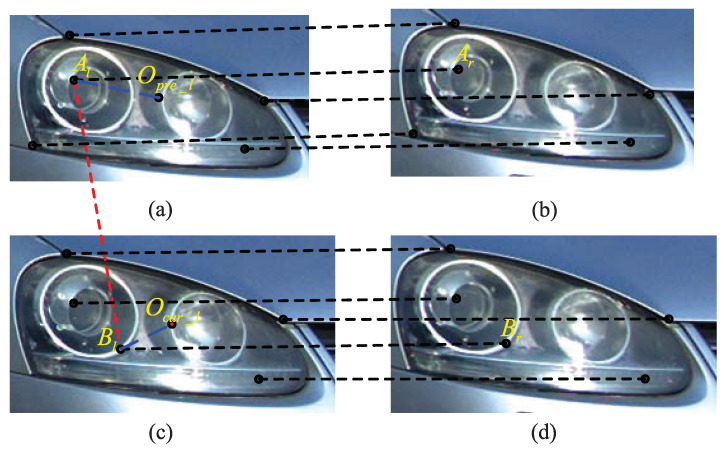
An exemplary result of speed measurement point selection by the method in [15]. (**a**) Previous left-view. (**b**) Previous right-view. (**c**) Current left-view. (**d**) Current right-view.

Figure 6 shows an exemplary result of speed measurement point selection by the method in [15]. In Figure 6, $O_{pre{,}_{-}l}$ is the center of the bounding box in the previous left-view frame, $O_{{cur}_{-}l}$ is the center of the bounding box in the current left-view frame, $A_{l}$ and $A_{r}$ are the selected speed measurement point pair in the previous stereo frames, $B_{l}$ and $B_{r}$ are the selected speed measurement point pair in the current stereo frames. The corresponding 3D speed measurement points *A* and *B* are obviously not on the same position of the vehicle, hence the displacement between *A* and *B* is not accurate for speed measurement.

**Figure 7:**
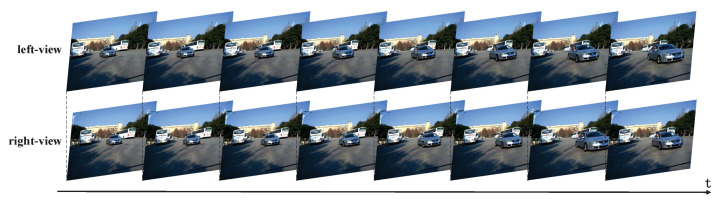
An exemplary stereo video sequence.

Figure 7 shows an exemplary stereo video sequence. The upper row is the time-continuous left-view video sequence, and the bottom row is the time-continuous right-view video sequence, either with temporal correlation [51]. Each column is a stereo image pair, with spatial correlation. Thus, stereo video sequence contains both spatial and temporal information, which should be fused to achieve more accurate speed measurement [52,53,54].

The matching point pair set obtained by SURF matching with LNCC-based mismatching removal on stereo frame pair is denoted as $S_{spa}={\left\{\left(p_{l_{-}i},p_{r_{-}i}\right)\right\}}_{i=1}^{M}$. The matching point pair set $S_{spa}$ of the current stereo frame pair is denoted as $S_{cur_{-}spa}={\left\{\left(p_{cur_{-}l_{-}i},p_{cur_{-}r_{-}i}\right)\right\}}_{i=1}^{M_{1}}$. The matching point pair set $S_{spa}$ of the previous stereo frame pair is denoted as $S_{pre_{-}spa}={\left\{\left(p_{pre_{-}l_{-}j},p_{pre_{-}r_{-}j}\right)\right\}}_{j=1}^{M_{2}}$. The matching point pair set obtained by SURF matching with LNCC-based mismatching removal on the previous and current left-view frames is denoted as $S_{temp}={\left\{\left(p_{l_{-}cur_{-}k},p_{l_{-}pre_{-}k}\right)\right\}}_{k=1}^{T}$. If $p_{l_{-}cur_{-}k}$ in the temporal matching point pair $\left(p_{l_{-}cur_{-}k},p_{l_{-}pre_{-}k}\right)$ is equal to $p_{cur_{-}l_{-}i}$ in the current spatial matching point pair $\left(p_{cur_{-}l_{-}i},p_{cur_{-}r_{-}i}\right)$, and if $p_{l_{-}pre_{-}k}$ in the temporal matching point pair $\left(p_{l_{-}cur_{-}k},p_{l_{-}pre_{-}k}\right)$ is equal to $p_{pre_{-}l_{-}j}$ in the previous spatial matching point pair $\left(p_{pre_{-}l_{-}j},p_{pre_{-}r_{-}j}\right)$, that is, $p_{l_{-}cur_{-}k}=p_{cur_{-}l_{-}i}$ & $p_{l_{-}pre_{-}k}=p_{pre_{-}l_{-}j}$, then it can be judged that $\left(p_{cur\_l\_i},p_{cur\_r\_i}\right)$ and $\left(p_{pre\_l\_j},p_{pre\_r\_j}\right)$ satisfy both SCC and TCC. All current $\left(p_{cur\_l\_i},p_{cur\_r\_i}\right)$ satisfying both SCC and TCC are placed in a new smaller matching point set $S_{spa\_temp}={\left\{\left(p_{cur\_l\_m},p_{cur\_r\_m}\right)\right\}}_{m=1}^{M_{3}}$, $S_{spa\_temp}\subset S_{cur\_spa}$. According to Equation (4), the distance $d_{m}$ between the left-view matching point $p_{cur\_l\_m}\left(x_{cur\_l\_m},y_{cur\_l\_m}\right)$ and the left-view bounding box center $O_{cur\_l}\left(x_{cur\_l},y_{cur\_l}\right)$ for each matching point pair in the set $S_{spa\_temp}$ is calculated:

(4) $$d_{m}=\sqrt{{\left(x_{cur\_l\_m}-x_{cur\_l}\right)}^{2}+{\left(y_{cur\_l\_m}-y_{cur\_l}\right)}^{2}}$$

The matching point pair with the minimum $d_{m}$ is selected as the optimum speed measurement point $\left(p_{cur\_l\_m_{opt}},p_{cur\_r\_m_{opt}}\right)$ for the current stereo frame pair:

(5) $$\left(p_{cur\_l\_m_{opt}},p_{cur\_r\_m_{opt}}\right)\in S_{spa\_temp},\;s.t.\;d_{m_{opt}}={\left\{d_{m}\right\}}_{\min}\;m=1,...,M_{3}$$

**Figure 8:**
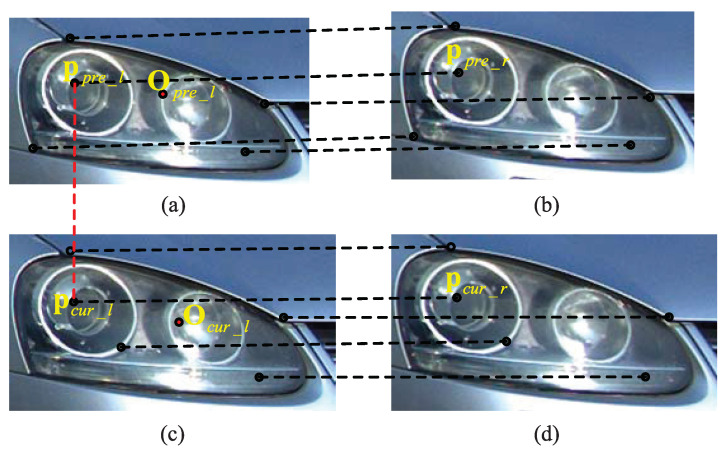
An exemplary result of speed measurement point selection by the proposed STIF-based method. (**a**) Previous left-view. (**b**) Previous right-view. (**c**) Current left-view. (**d**) Current right-view.

Algorithm 1 describes the optimum speed measurement point selection process based on STIF. Figure 8 shows an exemplary result of speed measurement point selection by the proposed STIF-based method. The corresponding 3D speed measurement points $p_{pre}$ and $p_{cur}$ are on the same position of the vehicle, hence the displacement between $p_{pre}$ and $p_{cur}$ is more accurate for speed measurement.**Algorithm 1:**Optimum speed measurement point selection based on STIF.**Input:** $S_{cur\_spa}$=${\left\{\left(p_{cur\_l\_i},p_{cur\_r\_i}\right)\right\}}_{i=1}^{M_{1}}$$S_{pre\_spa}$=${\left\{\left(p_{pre\_l\_j},p_{pre\_r\_j}\right)\right\}}_{j=1}^{M_{2}}$$S_{temp}$=${\left\{\left(p_{l\_cur\_k},p_{l\_pre\_k}\right)\right\}}_{k=1}^{T}$$O_{cur\_l}$$\left(x_{cur\_l},y_{cur\_l}\right)$**Output:** $\left(p_{cur\_l\_m_{opt}},p_{cur\_r\_m_{opt}}\right)$1:**function** Optimum speed measurement point selection 2:    **for** k=1 **to** *T* **do**3:        take $\left(p_{l\_cur\_k},p_{l\_pre\_k}\right)$∈$S_{temp}$4:        search $S_{cur\_spa}$5:        **if** $p_{l\_cur\_k}$=$p_{cur\_l\_i}$ **then**6:           search $S_{pre\_spa}$7:           **if** $p_{l\_pre\_k}$=$p_{pre\_l\_j}$ **then**8:               $S_{spa\_temp}$⟸$\left(p_{cur\_l\_i},p_{cur\_r\_i}\right)$9:           **end if**10:        **end if**11:    **end for**12:    **for** m=1 **to** size of $S_{spa\_temp}$ **do**13:        calculate $d_{m}$=$\sqrt{{\left(x_{cur\_l\_m}-x_{cur\_l}\right)}^{2}+{\left(y_{cur\_l\_m}-y_{cur\_l}\right)}^{2}}$14:    **end for**15:    select $d_{m_{opt}}$=${\left\{d_{m}\right\}}_{min}$16:    **return** $\left(p_{cur\_l\_m_{opt}},p_{cur\_r\_m_{opt\;}}\right)$ with $d_{m_{opt}}$17:**end function**

Table 5 shows the comparison of information entropy (IE) and normalized mutual information (NMI) with different constraints for license plate, logo, light and mirror, respectively. IE is used to measure the uncertainty of the matching point sets. The smaller the IE, the less the uncertainty. NMI is used to measure the similarity between the left-view and right-view matching point sets. The closer the NMI is to 1, the higher the similarity is, and the more accurate the matching point pair is. With LPSC, the IE of the left-view and right-view matching point sets is reduced, while the NMI thereof is increased. With LNCC, the IE of the left-view and right-view matching point sets is further reduced, while the NMI thereof is further increased. With STIF, the IE of the left-view and right-view matching point sets is even more reduced, while the NMI thereof is even more increased. The IE decreases and the NMI increases gradually with the increase of constraints, which indicates that the matching point pairs in the sets are becoming more accurate from the perspective of information entropy.

## 4. Experiments

In a practical vehicle speed measurement test, a fixed binocular stereo camera is set to capture images at a frame rate of 30 fps, and the vehicle speed is measured ten times per second. The speed data measured by GPS satellite speedometer is used as the ground truth for comparison. The vehicle drives towards the camera in a straight line at a constant speed. Six groups of experiments are conducted with different vehicle speed, i.e., 32 km/h, 36 km/h, 38 km/h, 43 km/h, 45 km/h and 46 km/h. For the captured stereo video of each experiment, the stereo matching algorithm in [15], the proposed LNCC stereo matching algorithm and the proposed LNCC+STIF stereo matching algorithm are, respectively, used to measure the vehicle speed, and the measured speed, error, root-mean-square error (RMSE), maximum absolute error (MAE) and maximum absolute error rate (MAER) of the three algorithms compared together. The algorithms are verified from three aspects: speed measurement results based on license plate, speed measurement results based on other separate vehicle characteristic, and speed measurement result based on multi-characteristic combination. Finally, the vehicle multi-characteristic combination-based speed measurement result by LNCC+STIF algorithm is compared with other speed measurement algorithms.

### 4.1. Speed Measurement Results Based on License Plate

**Figure 9:**
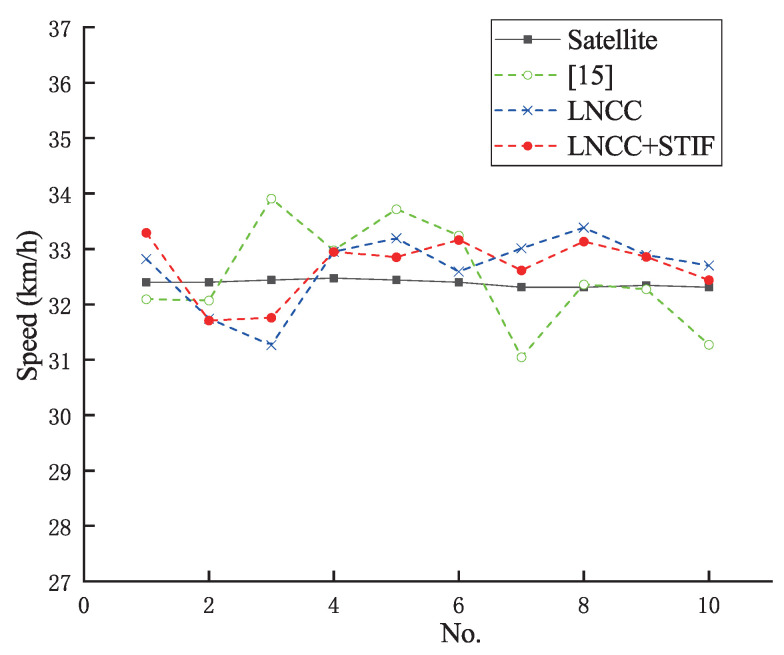
Speed measurement result curve based on license plate at a vehicle speed of 32 km/h.

First, the vehicle speed is measured using the license plate. Figure 9 shows the speed measurement result curve based on a license plate at a vehicle speed of 32 km/h. The black solid line represents the ground truth of vehicle speed measured by the satellite, the green dotted line with hollow circle represents the vehicle speed measurement results measured by the stereo matching algorithm in [15], the blue dotted line with cross represents the vehicle speed measurement results measured by the proposed LNCC stereo matching algorithm, and the red dotted line with solid circle represents the vehicle speed measurement results measured by the proposed LNCC+STIF stereo matching algorithm. As it can be seen from Figure 9, the vehicle speed measurement results based on license plate measured by the proposed LNCC+STIF stereo matching algorithm are closer to the ground truth speeds, with smaller fluctuations.

**Figure 10:**
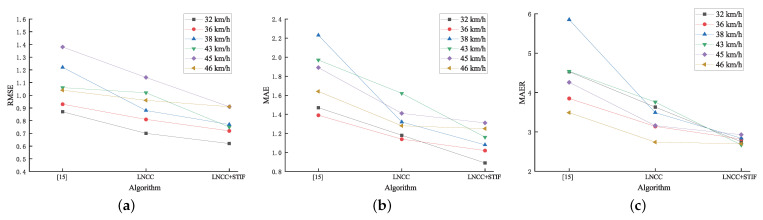
Error curve. (**a**) RMSE curve. (**b**) MAE curve. (**c**) MAER curve.

Table 6 shows the detailed speed measurement results based on license plate by the three algorithms at a vehicle speed of 32 km/h. The RMSE of the speeds measured by the stereo matching algorithm in [15], the LNCC stereo matching algorithm, the LNCC+STIF stereo matching algorithm is 0.87 km/h, 0.70 km/h and 0.62 km/h, respectively. The MAE of the speeds measured by the three algorithms is 1.47 km/h, 1.18 km/h and 0.89 km/h, respectively. The MAER of the speeds measured by the three algorithms is 4.53%, 3.63% and 2.75%, respectively. More experiments are carried out for the speed measurement by license plate. Table 7 shows the experimental error results at a vehicle speed of 36 km/h, 38 km/h, 43 km/h, 45 km/h and 46 km/h. As can be seen from Table 6 and Table 7, the speed measurement error results based on license plate by the three algorithms do not exceed the 6% error rate limit specified by the China national standard GB/T21255-2007 [55]. However, the speed measurement results based on license plate by the LNCC+STIF stereo matching algorithm have the least RMSE, MAE and MAER of the three. Therefore, the LNCC+STIF stereo matching algorithm effectively reduces the speed measurement error by license plate and enhances the measurement accuracy thereof. Figure 10a–c show the RMSE curve, the MAE curve and the MAER curve of the three algorithms, respectively. The curves uniformly show a descending trend.

### 4.2. Speed Measurement Results Based on Other Separate Vehicle Characteristic

Then, the vehicle speed is measured using other separate vehicle characteristics, i.e., logo, light and mirror. Table 8 shows the speed measurement error results based on logo, light and mirror by the three algorithms at a vehicle speed of 32 km/h. The RMSE of the logo-based speeds measured by the stereo matching algorithm in [15], the LNCC stereo matching algorithm, the LNCC+STIF stereo matching algorithm is 0.87 km/h, 0.79 km/h and 0.67 km/h, respectively. The MAE of the logo-based speeds measured by the three algorithms is 1.63 km/h, 1.18 km/h and 0.98 km/h. The MAER of the logo-based speeds measured by the three algorithms is 5.03%, 3.62% and 3.01%. The RMSE of the light-based speeds measured by the three algorithms is 1.03 km/h, 0.92 km/h and 0.71 km/h. The MAE of the light-based speeds measured by the three algorithms is 1.48 km/h, 1.46 km/h and 0.93 km/h. The MAER of the light-based speeds measured by the three algorithms is 4.57%, 4.49% and 2.89%. The RMSE of the mirror-based speeds measured by the three algorithms is 8.63 km/h, 1.32 km/h and 0.97 km/h. The MAE of the mirror-based speeds measured by the three algorithms is 19.07 km/h, 1.92 km/h and 1.85 km/h. The MAER of the mirror-based speeds measured by the three algorithms is 58.86%, 5.93% and 5.70%.

More experiments are carried out for the speed measurement by logo, light and mirror. Table 9 shows the experimental results at a vehicle speed of 36 km/h, 38 km/h, 43 km/h, 45 km/h and 46 km/h. As can be seen from Table 8 and Table 9, the speed measurement results based on logo and light by the three algorithms do not exceed the 6% error rate limit specified by the China national standard GB/T21255-2007 [55], but the speed measurement results based on mirror by the three algorithms are quite different. The mirror-based error rate by the stereo matching algorithm in [15] is much higher than the 6% error rate limit. The mirror-based error rate by the LNCC stereo matching algorithm also exceeds the 6% error rate limit. Only the mirror-based error rate by the LNCC+STIF stereo matching algorithm with the least RMSE, MAE and MAER satisfies the 6% error rate limit. Therefore, the LNCC+STIF stereo matching algorithm effectively reduces the speed measurement error by logo, light and mirror, and enhances the measurement accuracy thereof.

### 4.3. Speed Measurement Results Based on Multi-Characteristic Combination

Finally, to further reduce the error based on single-characteristic, the speed measurement results of license plate, logo, light and mirror by the proposed LNCC+STIF stereo matching algorithm are averaged as the final speed measurement results based on multi-characteristic combination.

**Figure 11:**
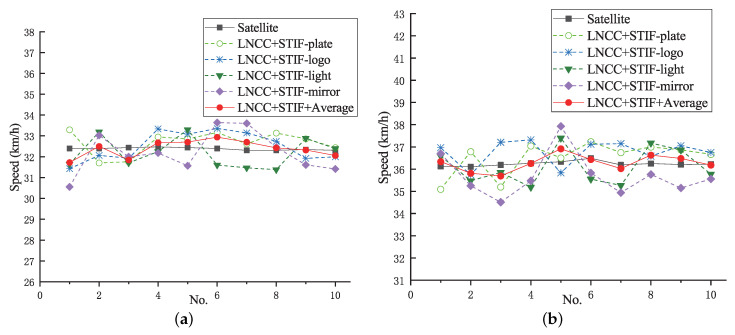
Speed measurement result curve by the proposed LNCC+STIF at a vehicle speed. (**a**) 32 km/h. (**b**) 36 km/h.

Figure 11 shows the speed measurement result curve by the proposed LNCC+STIF stereo matching algorithm at a vehicle speed of 32 km/h and 36 km/h, respectively. The black solid line with square represents the ground truth of vehicle speed measured by the satellite, the red solid line with circle represents the average speed results, the green dotted line with a hollow circle represents the speed results based on license plate, the blue dotted line with cross represents the speed results based on logo, the green dotted line with triangle represents the speed results based on light, and the purple dotted line with diamond represents the speed results based on mirror. As it can be seen from Figure 11a,b, the vehicle speed measurement results based on multi-characteristic combination measured by the proposed LNCC+STIF stereo matching algorithm are closer to the ground truth speeds, with smaller fluctuations.

Table 10 shows the detailed speed measurement results by the proposed LNCC+STIF algorithm at a vehicle speed of 32 km/h. The RMSE of the speeds measured based on license plate, logo, light, mirror and average is 0.62 km/h, 0.67 km/h, 0.71 km/h, 0.97 km/h and 0.38 km/h, respectively. The MAE of the speeds measured based on license plate, logo, light, mirror and average is 0.89 km/h, 0.98 km/h, 0.93 km/h, 1.85 km/h and 0.67 km/h, respectively. The MAER of the speeds measured based on license plate, logo, light, mirror and average is 2.75%, 3.01%, 2.89%, 5.70% and 2.08%, respectively. More experiments are carried out for the speed measurement by the proposed LNCC+STIF algorithm. Table 11 shows the experimental error results at a vehicle speed of 36 km/h, 38 km/h, 43 km/h, 45 km/h and 46 km/h. As can be seen from Table 10 and Table 11, the speed measurement error results based on license plate, logo, light, mirror and average by the proposed LNCC+STIF algorithm do not exceed the 6% error rate limit. However, the speed measurement results based on average have the least RMSE, MAE and MAER of the five. Therefore, the LNCC+STIF stereo matching algorithm based on average effectively reduces the speed measurement error and enhances the measurement accuracy, which is chosen as the optimum stereo matching algorithm for the vehicle speed measurement system.

Meanwhile, the speed measurement performances are compared between the system with the proposed optimum stereo matching algorithm and the various existing speed measurement systems. Table 12 shows a comparison of the speed measurement error results between the proposed system and the other four systems. The systems in [11,56] are 2D video-based speed measurement which are only suitable for measuring the speed of vehicle traveling in a straight line and are not accurate enough. The systems in [13,15] are 3D video-based speed measurement, which are suitable for measuring the speed of vehicle traveling in a straight or curved line. However, the stereo matching in [13,15] is simple and rough, which may lead to inaccurate speed measurement as well. The proposed system improves the stereo matching with LNCC and STIF, which results in more accurate speed measurement. It can be seen that the RMSE of the proposed system is smaller than that of the other four systems, and the maximum error is also smaller than that of the other four systems. Therefore, the speed measurement accuracy of the proposed system is superior to that of the other four systems, that is, the speed measurement accuracy of the system is improved.

## 5. Conclusions

In this study, we improved the stereo matching algorithm for vehicle speed measurement system based on binocular stereovision. We first proposed a mismatching removal algorithm based on LPSC for the license plate. We then proposed a mismatching removal algorithm based on LNCC for multiple characteristics of the vehicle. We finally proposed a speed measurement point selection algorithm based on STIF. We combined LNCC with STIF to further improve the stereo matching algorithm. Vehicle speed measurement experiments were carried out by three stereo matching algorithms and the results were compared, based on license plate and other separate vehicle characteristic, respectively. Experimental results demonstrate that the proposed LNCC+STIF stereo matching algorithm can efficiently enhance the speed measurement accuracy. Vehicle speed measurement experiments based on license plate, logo, light, mirror and average were also carried out by the proposed LNCC+STIF stereo matching algorithm. Experimental results demonstrate that the proposed LNCC+STIF stereo matching algorithm based on average can further improve the speed measurement accuracy. Performance comparisons were made between the system with the proposed optimum stereo matching algorithm and the various existing speed measurement systems, which demonstrates that the vehicle speed measurement system with the proposed optimum stereo matching algorithm can significantly outperform the state-of-the-art system in accuracy.

## Figures and Tables

**Table 1:** The pixel ratio of the license plate in the image captured at different distance.

No.	Distance (m)	Pixel Ratio (%)	No.	Distance (m)	Pixel Ratio (%)	No.	Distance (m)	Pixel Ratio (%)
1	15.0	0.0416	11	10.0	0.0913	21	5.0	0.3372
2	14.5	0.0470	12	9.5	0.1000	22	4.5	0.4095
3	14.0	0.0482	13	9.0	0.1134	23	4.0	0.5180
4	13.5	0.0523	14	8.5	0.1206	24	3.5	0.6807
5	13.0	0.0553	15	8.0	0.1390	25	3.0	0.9524
6	12.5	0.0614	16	7.5	0.1580	26	2.5	1.3669
7	12.0	0.0646	17	7.0	0.1728	27	2.0	2.1482
8	11.5	0.0694	18	6.5	0.2008	28	1.5	3.9328
9	11.0	0.0750	19	6.0	0.2343	29	1.0	8.1130
10	10.5	0.0830	20	5.5	0.2747			

**Table 2:** Performance comparison of different fitting functions.

Fitting Function	RMSE	SSE	R-Square	Adj R-sq
Polynomial-7	0.092	0.152	0.999	0.999
Polynomial-8	0.067	0.076	0.999	0.999
Power-1	0.117	0.326	0.999	0.999
Power-2	0.100	0.230	0.999	0.999

**Table 3:** Comparison of matching point pair number with and without LPSC.

No.	1	2	3	4	5	6	7	8	9	10
SURF	84	123	149	160	184	238	264	288	324	665
SURF with LPSC	35	26	35	54	60	69	106	105	106	249

**Table 4:** Comparison of matching point pair number with and without LNCC.

No.		1	2	3	4	5	6	7	8	9	10
License plate	SURF	84	123	149	160	184	238	264	288	324	665
	SURF with LNCC	18	14	33	160	38	53	98	67	58	237
Logo	SURF	36	40	46	65	84	95	104	114	137	169
	SURF with LNCC	26	29	30	38	47	55	74	60	79	103
Light	SURF	73	96	119	146	193	283	355	459	294	694
	SURF with LNCC	33	77	88	113	141	197	234	312	195	403
Mirror	SURF	26	27	29	29	36	48	48	50	79	99
	SURF with LNCC	18	20	23	25	28	29	39	31	51	65

**Table 5:** Comparison of information entropy and NMI with different constraints.

	Constraint	IE (Bit/Pixel)		NMI
		Left-View	Right-View	
License plate	SURF	3.5609	3.5549	0.7570
	SURF with LPSC	3.1498	3.3349	0.8959
	SURF with LPSC and LNCC	3.1680	3.3779	0.8959
	SURF with LPSC, LNCC and STIF	2.7170	2.8372	0.9082
Logo	SURF	5.0988	4.7373	0.7966
	SURF with LNCC	4.7376	4.6422	0.8725
	SURF with LNCC and STIF	3.1219	3.1219	0.9359
Light	SURF	5.8604	5.6467	0.8080
	SURF with LNCC	5.6615	5.3441	0.8430
	SURF with LNCC and STIF	5.1881	5.0897	0.8991
Mirror	SURF	3.2924	2.9693	0.9085
	SURF with LNCC	2.4619	2.4619	1.0000
	SURF with LNCC and STIF	1.9219	1.9219	1.0000

**Table 6:** Speed measurement results based on license plate at a vehicle speed of 32 km/h.

No.	Satellite (km/h)			LNCC		LNCC+STIF	
		Speed (km/h)	Error (km/h)	Speed (km/h)	Error (km/h)	Speed (km/h)	Error (km/h)
1	32.40	32.10	−0.30	32.82	0.42	33.29	0.89
2	32.40	32.07	−0.33	31.74	−0.66	31.71	−0.69
3	32.44	33.91	1.47	31.26	−1.18	31.76	−0.68
4	32.47	32.98	0.51	32.95	0.48	32.95	0.48
5	32.44	33.72	1.28	33.19	0.75	32.85	0.41
6	32.40	33.24	0.84	32.59	0.19	33.16	0.76
7	32.31	31.05	−1.26	33.01	0.70	32.62	0.31
8	32.31	32.36	0.05	33.39	1.08	33.14	0.83
9	32.34	32.27	−0.07	32.89	0.55	32.86	0.52
10	32.31	31.27	−1.04	32.70	0.39	32.44	0.13
RMSE			0.87		0.70		0.62
MAE			1.47		1.18		0.89
MAER			4.53%		3.63%		2.75%

**Table 7:** Speed measurement error results based on license plate.

No.	36 km/h			38 km/h			43 km/h			45 km/h			46 km/h		
	(km/h)	LNCC (km/h)	LNCC+STIF (km/h)	(km/h)	LNCC (km/h)	LNCC+STIF (km/h)	(km/h)	LNCC (km/h)	LNCC+STIF (km/h)	(km/h)	LNCC (km/h)	LNCC+STIF (km/h)	(km/h)	LNCC (km/h)	LNCC+STIF (km/h)
1	−0.09	0.10	−1.02	1.79	−0.67	−0.95	0.15	−1.62	0.39	−1.89	1.40	−0.94	−1.14	1.03	−1.08
2	1.39	0.79	0.68	−0.16	0.26	−0.91	1.97	1.01	1.16	−1.09	−0.63	−1.31	−0.34	−1.04	−1.04
3	0.29	−0.71	−0.99	1.31	0.59	0.53	−0.98	−1.36	0.80	1.87	1.41	0.93	0.70	−0.69	−1.25
4	−0.74	−1.14	0.78	0.35	−1.04	−0.56	1.10	1.35	−0.57	−1.07	−0.24	−0.84	0.47	−1.12	0.91
5	−0.75	1.00	0.17	1.63	1.32	0.77	−0.46	−0.75	1.10	−0.08	−0.85	−1.24	0.70	0.32	−1.23
6	1.01	0.38	0.74	−0.13	1.02	−0.68	−0.48	−0.44	0.51	1.07	1.40	1.09	0.66	−1.19	−0.70
7	1.21	−0.91	0.56	2.23	−0.33	1.08	1.56	−0.93	0.55	1.26	−1.33	0.76	0.23	−1.28	0.58
8	−0.80	0.95	0.74	0.17	0.75	−0.54	0.99	−0.55	−0.93	−1.89	0.91	0.72	−1.59	0.71	−0.51
9	−0.70	0.77	0.65	1.44	1.24	0.63	0.18	−1.04	0.60	−1.63	−1.39	0.46	−1.64	0.62	−1.00
10	1.37	0.76	0.44	0.37	0.89	0.87	−1.08	−0.39	0.44	0.77	1.18	0.25	−1.56	0.80	−0.08
RMSE	0.93	0.81	0.72	1.22	0.88	0.77	1.06	1.02	0.75	1.38	1.14	0.91	1.04	0.93	0.91
MAE	1.39	1.14	1.02	2.23	1.32	1.08	1.97	1.62	1.16	1.89	1.41	1.31	1.64	1.28	1.25
MAER	3.85%	3.14%	2.82%	5.85%	3.49%	2.84%	4.54%	3.76%	2.67%	4.26%	3.16%	2.93%	3.49%	2.74%	2.70%

**Table 8:** Speed measurement error results based on logo, light and mirror at a vehicle speed of 32 km/h.

No.	Logo			Light			Mirror		
	(km/h)	LNCC (km/h)	LNCC+STIF (km/h)	(km/h)	LNCC (km/h)	LNCC+STIF (km/h)	(km/h)	LNCC (km/h)	LNCC+STIF (km/h)
1	−0.30	−0.98	−0.98	0.59	−1.08	−0.76	19.07	−1.85	−1.85
2	0.64	−0.70	−0.34	1.48	1.46	0.79	−2.22	0.63	0.63
3	1.63	1.18	−0.51	−0.40	0.23	−0.73	−11.25	1.01	−0.44
4	0.10	0.63	0.86	0.24	0.51	−0.22	6.53	0.65	−0.29
5	0.81	1.12	0.64	1.43	0.84	0.86	−5.42	−1.39	−0.87
6	0.61	0.33	0.96	0.68	−1.24	−0.80	0.05	−1.92	1.24
7	−0.89	−0.94	0.83	−0.01	0.73	−0.84	3.21	−0.79	1.29
8	1.52	−0.56	0.42	−1.25	0.49	−0.93	9.25	1.39	0.15
9	−0.06	0.47	−0.42	1.34	1.20	0.54	−8.93	−0.65	−0.73
10	−0.59	−0.46	−0.31	1.40	0.70	0.08	1.37	−1.87	−0.90
RMSE	0.87	0.79	0.67	1.03	0.92	0.71	8.63	1.32	0.97
MAE	1.63	1.18	0.98	1.48	1.46	0.93	19.07	1.92	1.85
MAER	5.03%	3.62%	3.01%	4.57%	4.49%	2.89%	58.86%	5.93%	5.70%

**Table 9:** Speed measurement error results based on logo, light and mirror.

Speed	Parameter	Logo			Light			Mirror		
		(km/h)	LNCC (km/h)	LNCC+STIF (km/h)	(km/h)	LNCC (km/h)	LNCC+STIF (km/h)	(km/h)	LNCC (km/h)	LNCC+STIF (km/h)
36 km/h	RMSE	0.85	0.81	0.75	1.13	0.89	0.79	4.89	1.43	1.04
	MAE	1.29	1.18	1.06	1.92	1.33	1.07	11.18	2.19	1.68
	MAER	3.57%	3.26%	2.91%	5.30%	3.66%	2.96%	30.96%	5.99%	4.65%
38 km/h	RMSE	1.01	0.91	0.87	1.15	1.06	0.81	13.48	1.48	1.06
	MAE	1.49	1.31	1.10	1.82	1.70	1.11	19.79	2.30	1.74
	MAER	3.97%	3.44%	2.87%	4.86%	4.45%	2.92%	52.71%	6.20%	4.54%
43 km/h	RMSE	1.05	0.95	0.90	1.39	1.12	0.79	40.57	1.39	1.23
	MAE	1.62	1.58	1.14	2.45	1.76	1.12	88.03	2.35	2.35
	MAER	3.71%	3.64%	2.63%	5.66%	4.06%	2.57%	201.53%	5.41%	5.41%
45 km/h	RMSE	1.04	1.26	0.91	1.30	1.15	0.98	10.10	1.72	1.50
	MAE	1.97	1.88	1.24	2.17	1.80	1.30	21.76	2.60	2.55
	MAER	4.38%	4.17%	2.75%	4.84%	4.02%	2.88%	48.25%	5.80%	5.66%
46 km/h	RMSE	1.35	1.23	0.86	1.39	1.01	0.89	27.25	2.05	1.54
	MAE	2.33	1.69	1.39	2.79	1.58	1.31	60.93	3.24	2.72
	MAER	5.01%	3.62%	2.96%	5.97%	3.39%	2.80%	130.88%	6.94%	5.92%

**Table 10:** Speed measurement results by the proposed LNCC+STIF algorithm at a vehicle speed of 32 km/h.

No.	Satellite (km/h)	Plate		Logo		Light		Mirror		Average	
		Speed (km/h)	Error (km/h)	Speed (km/h)	Error (km/h)	Speed (km/h)	Error (km/h)	Speed (km/h)	Error (km/h)	Speed (km/h)	Error (km/h)
1	32.40	33.29	0.89	31.42	−0.98	31.64	−0.76	30.55	−1.85	31.73	−0.67
2	32.40	31.71	−0.69	32.06	−0.34	33.19	0.79	33.03	0.63	32.50	0.10
3	32.44	31.76	−0.68	31.93	−0.51	31.71	−0.73	32.00	−0.44	31.85	−0.59
4	32.47	32.95	0.48	33.33	0.86	32.25	−0.22	32.18	−0.29	32.68	0.21
5	32.44	32.85	0.41	33.08	0.64	33.30	0.86	31.57	−0.87	32.70	0.26
6	32.40	33.16	0.76	33.36	0.96	31.60	−0.80	33.64	1.24	32.94	0.54
7	32.31	32.62	0.31	33.14	0.83	31.47	−0.84	33.60	1.29	32.71	0.40
8	32.31	33.14	0.83	32.73	0.42	31.38	−0.93	32.46	0.15	32.43	0.12
9	32.34	32.86	0.52	31.92	−0.42	32.88	0.54	31.61	−0.73	32.32	−0.02
10	32.31	32.44	0.13	32.00	−0.31	32.39	0.08	31.41	−0.90	32.06	−0.25
RMSE			0.62		0.67		0.71		0.97		0.38
MAE			0.89		0.98		0.93		1.85		0.67
MAER			2.75%		3.01%		2.89%		5.70%		2.08%

**Table 11:** Speed measurement error results by the proposed LNCC+STIF algorithm.

Speed	Parameter	Plate (km/h)	Logo (km/h)	Light (km/h)	Mirror (km/h)	Average (km/h)
36 km/h	RMSE	0.72	0.75	0.79	1.04	0.30
	MAE	1.02	1.06	1.07	1.68	0.54
	MAER	2.82%	2.91%	2.96%	4.65%	1.48%
38 km/h	RMSE	0.77	0.87	0.81	1.06	0.54
	MAE	1.08	1.10	1.11	1.74	1.06
	MAER	2.84%	2.87%	2.92%	4.54%	2.79%
43 km/h	RMSE	0.75	0.90	0.79	1.23	0.37
	MAE	1.16	1.14	1.12	2.35	0.77
	MAER	2.67%	2.63%	2.57%	5.41%	1.77%
45 km/h	RMSE	0.91	0.91	0.98	1.50	0.40
	MAE	1.31	1.24	1.30	2.55	0.90
	MAER	2.93%	2.75%	2.88%	5.66%	1.99%
46 km/h	RMSE	0.91	0.86	0.89	1.54	0.43
	MAE	1.25	1.39	1.31	2.72	0.84
	MAER	2.70%	2.96%	2.80%	5.92%	1.79%

**Table 12:** Comparison of speed measurement error results among different vehicle speed measurement systems.

System	RMSE (km/h)	Max Error (km/h)
Luvizo et al. [11]	1.36	[−4.68,+6.00]
Tang et al. [56]	6.59	NA
VSS-SURF [13]	1.29	[−2.0,+2.0]
Yang et al. [15]	0.65	[−1.6,+1.1]
Proposed	0.40	[−0.9,+1.06]

## Data Availability

Not applicable.

## References

[1] Choy J.L.C., Wu J., Long C., Lin Y.B. (2020). Ubiquitous and Low Power Vehicles Speed Monitoring for Intelligent Transport Systems. IEEE Sens. J..

[2] Shin H.S., Turchi D., He S., Tsourdos A. (2019). Behavior Monitoring Using Learning Techniques and Regular-Expressions-Based Pattern Matching. IEEE Trans. Intell. Transp. Syst..

[3] Zhang C., Ota K., Jia J., Dong M. (2018). Breaking the Blockage for Big Data Transmission: Gigabit Road Communication in Autonomous Vehicles. IEEE Commun. Mag..

[4] Balid W., Tafish H., Refai H.H. (2018). Intelligent Vehicle Counting and Classification Sensor for Real-Time Traffic Surveillance. IEEE Trans. Intell. Transp. Syst..

[5] Wei Q., Yang B. (2017). Adaptable Vehicle Detection and Speed Estimation for Changeable Urban Traffic with Anisotropic Magnetoresistive Sensors. IEEE Sens. J..

[6] Makarov A. (2016). Real-Time Vehicle Speed Estimation Based on License Plate Tracking in Monocular Video Sequences. Sens. Transducers.

[7] Ki Y.K., Baik D.K. (2006). Model for accurate speed measurement using double-loop detectors. IEEE Trans. Veh. Technol..

[8] Odat E., Shamma J.S., Claudel C. (2018). Vehicle Classification and Speed Estimation Using Combined Passive Infrared/Ultrasonic Sensors. IEEE Trans. Intell. Transp. Syst..

[9] Quang V.V., Linh N.V., Thang V.T., Phuc D.V. (2020). Vehicle speed estimation using two roadside passive infrared sensors. Int. J. Mod. Phys. B.

[10] Jeng S.L., Chieng W.H., Lu H.P. (2014). Estimating Speed Using a Side-Looking Single-Radar Vehicle Detector. IEEE Trans. Intell. Transp. Syst..

[11] Luvizon D.C., Nassu B.T., Minetto R. (2017). A Video-Based System for Vehicle Speed Measurement in Urban Roadways. IEEE Trans. Intell. Transp. Syst..

[12] Famouri M., Azimifar Z., Wong A. (2019). A Novel Motion Plane-Based Approach to Vehicle Speed Estimation. IEEE Trans. Intell. Transp. Syst..

[13] El Bouziady A., Thami R.O.H., Ghogho M., Bourja O., El Fkihi S. Vehicle speed estimation using extracted SURF features from stereo images. Proceedings of the 2018 International Conference on Intelligent Systems and Computer Vision (ISCV).

[14] Najman P., Zemčík P. (2020). Vehicle Speed Measurement Using Stereo Camera Pair. IEEE Trans. Intell. Transp. Syst..

[15] Yang L., Li M., Song X., Xiong Z., Hou C., Qu B. (2019). Vehicle Speed Measurement Based on Binocular Stereovision System. IEEE Access.

[16] Liu Y., Dominicis L.D., Wei B., Chen L., Martin R.R. (2015). Regularization Based Iterative Point Match Weighting for Accurate Rigid Transformation Estimation. IEEE Trans. Vis. Comput. Graph..

[17] Ma T., Ma J., Yu K., Zhang J., Fu W. (2021). Multispectral Remote Sensing Image Matching via Image Transfer by Regularized Conditional Generative Adversarial Networks and Local Feature. IEEE Geosci. Remote Sens. Lett..

[18] Ghaffari A., Fatemizadeh E. (2018). Image Registration Based on Low Rank Matrix: Rank-Regularized SSD. IEEE Trans. Med. Imaging.

[19] Liu X., Jing W., Zhou M., Li Y. (2019). Multi-Scale Feature Fusion for Coal-Rock Recognition Based on Completed Local Binary Pattern and Convolution Neural Network. Entropy.

[20] Ilyas A., Farid M.S., Khan M.H., Grzegorzek M. (2021). Exploiting Superpixels for Multi-Focus Image Fusion. Entropy.

[21] Leng C., Zhang H., Li B., Cai G., Pei Z., He L. (2019). Local Feature Descriptor for Image Matching: A Survey. IEEE Access.

[22] Jiang X., Ma J., Xiao G., Shao Z., Guo X. (2021). A review of multimodal image matching: Methods and applications. Inf. Fusion.

[23] Ma J., Ma Y., Li C. (2019). Infrared and visible image fusion methods and applications: A survey. Inf. Fusion.

[24] Reddy B., Chatterji B. (1996). An FFT-based technique for translation, rotation, and scale-invariant image registration. IEEE Trans. Image Process..

[25] Rangarajan A., Chui H., Duncan J.S. (2000). Rigid point feature registration using mutual information. Med. Image Anal..

[26] Liu X., Wang Z., Wang L., Huang C., Luo X. (2021). A Hybrid Rao-NM Algorithm for Image Template Matching. Entropy.

[27] Ma J., Zhao J., Tian J., Yuille A.L., Tu Z. (2014). Robust Point Matching via Vector Field Consensus. IEEE Trans. Image Process..

[28] Ma J., Jiang X., Fan A., Jiang J., Yan J. (2021). Image Matching from Handcrafted to Deep Features: A Survey. Int. J. Comput. Vis..

[29] Xu X., Yu C., Zhou J. Robust feature point matching based on geometric consistency and affine invariant spatial constraint. Proceedings of the 2013 IEEE International Conference on Image Processing.

[30] Kim J., Liu C., Sha F., Grauman K. Deformable Spatial Pyramid Matching for Fast Dense Correspondences. Proceedings of the 2013 IEEE Conference on Computer Vision and Pattern Recognition.

[31] Lowe D.G. (2004). Distinctive Image Features from Scale-Invariant Keypoints. Int. J. Comput. Vis..

[32] Bay H., Ess A., Tuytelaars T., Gool L.V. (2008). Speeded-Up Robust Features (SURF). Comput. Vis. Image Underst..

[33] Gao Z., Wang L., Zhou L. (2019). A Probabilistic Approach to Cross-Region Matching-Based Image Retrieval. IEEE Trans. Image Process..

[34] Qu H.B., Wang J.Q., Li B., Yu M. (2017). Probabilistic Model for Robust Affine and Non-Rigid Point Set Matching. IEEE Trans. Pattern Anal. Mach. Intell..

[35] Wu Y., Ma W., Gong M., Su L., Jiao L. (2015). A Novel Point-Matching Algorithm Based on Fast Sample Consensus for Image Registration. IEEE Geosci. Remote Sens. Lett..

[36] Torr P.H.S., Zisserman A. (2000). MLESAC: A New Robust Estimator with Application to Estimating Image Geometry. Comput. Vis. Image Underst..

[37] Chum O., Matas J. Matching with PROSAC—Progressive sample consensus. Proceedings of the 2005 IEEE Computer Society Conference on Computer Vision and Pattern Recognition (CVPR’05).

[38] Tuytelaars T., Mikolajczyk K. (2007). Local Invariant Feature Detectors. Found. Trends Comput. Graph. Vis..

[39] Gay-Bellile V., Bartoli A., Sayd P. (2010). Direct Estimation of Nonrigid Registrations with Image-Based Self-Occlusion Reasoning. IEEE Trans. Pattern Anal. Mach. Intell..

[40] Ma J., Qiu W., Zhao J., Ma Y., Yuille A.L., Tu Z. (2015). Robust *L*_2_*E* Estimation of Transformation for Non-Rigid Registration. IEEE Trans. Signal Process..

[41] Ma J., Zhao J., Jiang J., Zhou H., Guo X. (2019). Locality Preserving Matching. Int. J. Comput. Vis..

[42] Ma J., Jiang J., Zhou H., Zhao J., Guo X. (2018). Guided Locality Preserving Feature Matching for Remote Sensing Image Registration. IEEE Trans. Geosci. Remote Sens..

[43] Jiang X., Ma J., Jiang J., Guo X. (2020). Robust Feature Matching Using Spatial Clustering with Heavy Outliers. IEEE Trans. Image Process..

[44] Kuppala K., Banda S., Barige T.R. (2020). An overview of deep learning methods for image registration with focus on feature-based approaches. Int. J. Image Data Fusion.

[45] Ma J., Wu J., Zhao J., Jiang J., Zhou H., Sheng Q.Z. (2019). Nonrigid Point Set Registration with Robust Transformation Learning Under Manifold Regularization. IEEE Trans. Neural Netw..

[46] Ma J., Jiang X., Jiang J., Zhao J., Guo X. (2019). LMR: Learning a Two-Class Classifier for Mismatch Removal. IEEE Trans. Image Process..

[47] Liying Y.Y. (2014). License plates of motor vehicles of the People’s Republic of China. China National Standard GA 36—2014.

[48] Cai Z., Lan T., Zheng C. (2017). Hierarchical MK Splines: Algorithm and Applications to Data Fitting. IEEE Trans. Multimed..

[49] Wang X. (2018). Research on Target Ranging Technology Based on Binocular Stereo Vision. Master’s Thesis.

[50] Zhang Z. (2000). A flexible new technique for camera calibration. IEEE Trans. Pattern Anal. Mach. Intell..

[51] Yi P., Wang Z., Jiang K., Jiang J., Lu T., Ma J. (2020). A Progressive Fusion Generative Adversarial Network for Realistic and Consistent Video Super-Resolution. IEEE Trans. Pattern Anal. Mach. Intell..

[52] Pan Z., Yu W., Lei J., Ling N., Kwong S. (2021). TSAN: Synthesized View Quality Enhancement via Two-Stream Attention Network for 3D-HEVC. IEEE Trans. Circuits Syst. Video Technol..

[53] Usman M.A., Usman M.R., Shin S.Y. (2018). Exploiting the Spatio-Temporal Attributes of HD Videos: A Bandwidth Efficient Approach. IEEE Trans. Circuits Syst. Video Technol..

[54] Peng B., Lei J., Fu H., Jia Y., Zhang Z., Li Y. (2021). Deep video action clustering via spatio-temporal feature learning. Neurocomputing.

[55] Zhou C.Y. (2007). Motor Vehicle Speed Detector. China National Standard GB/T 21255-2007.

[56] Tang Z., Wang G., Xiao H., Zheng A., Hwang J.N. Single-Camera and Inter-Camera Vehicle Tracking and 3D Speed Estimation Based on Fusion of Visual and Semantic Features. Proceedings of the 2018 IEEE/CVF Conference on Computer Vision and Pattern Recognition Workshops (CVPRW).

